# Sugar, Gravel, Fish, and Flowers: Dependence of Mesoscale Patterns of Trade‐Wind Clouds on Environmental Conditions

**DOI:** 10.1029/2019GL085988

**Published:** 2020-03-26

**Authors:** Sandrine Bony, Hauke Schulz, Jessica Vial, Bjorn Stevens

**Affiliations:** ^1^ LMD/IPSL, CNRS, Sorbonne University Paris France; ^2^ Max Planck Institute for Meteorology Hamburg Germany

**Keywords:** trade‐wind clouds, mesoscale organization, shallow convection, low‐cloud feedback

## Abstract

Trade‐wind clouds exhibit a large diversity of spatial organizations at the mesoscale. Over the tropical western Atlantic, a recent study has visually identified four prominent mesoscale patterns of shallow convection, referred to as flowers, fish, gravel, and sugar. We show that these four patterns can be identified objectively from satellite observations by analyzing the spatial distribution of infrared brightness temperatures. By applying this analysis to 19 years of data, we examine relationships between cloud patterns and large‐scale environmental conditions. This investigation reveals that on daily and interannual timescales, the near‐surface wind speed and the strength of the lower‐tropospheric stability discriminate the occurrence of the different organization patterns. These results, combined with the tight relationship between cloud patterns, low‐level cloud amount, and cloud‐radiative effects, suggest that the mesoscale organization of shallow clouds might change under global warming. The role of shallow convective organization in determining low‐cloud feedback should thus be investigated.

## Key Points

- Prominent mesoscale patterns of shallow convection are identified from satellite observations
- Mesoscale patterns exhibit strong relationships with surface wind speed and lower‐tropospheric stability
- Owing to their differences in low‐cloud fraction, mesoscale patterns exert different impacts on the top‐of‐atmosphere radiation budget

## 1 Introduction

Shallow cumuli are ubiquitous over the world ocean, and therefore their sensitivity to a change in environmental conditions has the potential to greatly influence Earth's radiation balance and climate sensitivity. Actually, the response of trade‐wind cumuli to warming constitutes a primary source of uncertainty in climate model estimates of cloud feedbacks (Bony & Dufresne, 2005; Medeiros et al., 2015; Vial et al., 2013). During the last decade, much progress has been made in understanding the mechanisms through which trade‐wind cumuli could respond to warming (Bretherton, 2015; Klein et al., 2017; Rieck et al., 2012; Vogel et al., 2016). However, despite having long recognized that shallow convective clouds are patterned—or organized—on the mesoscale in many different ways (Agee, 1987; Malkus & Riehl, 1964), the role that this organization might play in cloud feedbacks remains largely unexplored (Nuijens & Siebesma, 2019; Vial et al., 2017). Thus, it remains an open question as to whether the mesoscale organization of clouds, which is left out of most parameterizations and the many large‐eddy simulations over small domains, influences how shallow convective clouds respond to warming.

**Figure 1:**
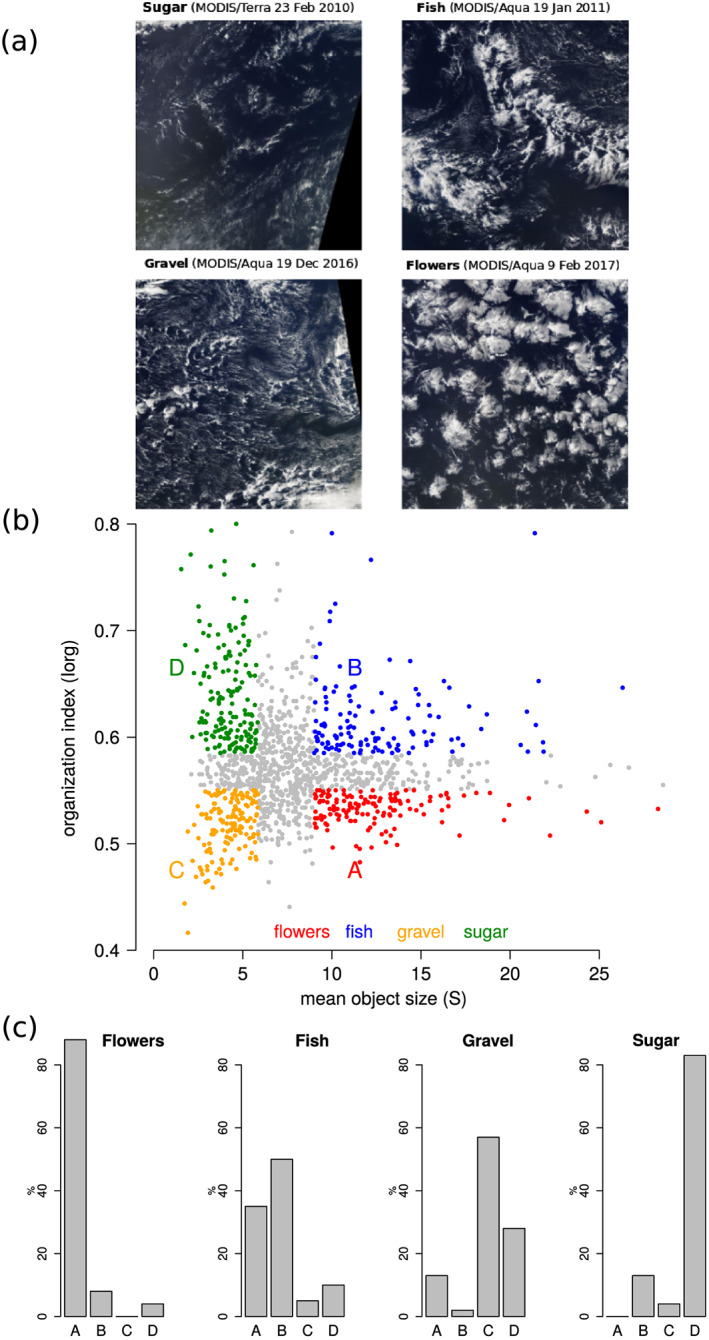
(a) Illustration of the four prominent cloud patterns of shallow convective organization pointed out by Stevens et al. (2019) over the tropical western Atlantic near Barbados. The four satellite images (48–58° W, 10–20° N) are derived from MODIS imagery. (b) Characterization of the shallow convective organization using infrared geostationary satellite data through two metrics: a convective organization index (I ${}_{org}$) and the mean object size ( S). The lower and upper terciles of I ${}_{org}$ and S define four classes of mesoscale organization (Quadrants A, B, C, and D). (c) Relative occurrence of the four cloud patterns defined by Stevens et al. (2019) in each quadrant of the (S, I ${}_{org}$) distribution.

To help answer this question, here we first explore whether the observed organization of shallow convection can be linked to variability in large‐scale environmental conditions and whether the different patterns of organization imprint themselves differently on the radiation budget. We do so by using observations over a region of the North Atlantic trades (windward of Barbados) where the shallow clouds are known to be representative of the broader trades (Medeiros & Nuijens, 2016) and where the mesoscale organization of shallow clouds has been well characterized (Stevens et al., 2019). During boreal winter, this region is associated with sea surface temperatures (SSTs) of 26 to 28 ° C, a moderate large‐scale subsidence in the free troposphere (about 25 to 30 hPa $d^{-1}$) and a predominance of shallow clouds (Stevens et al., 2016). In this region, the prominent patterns of organization do not correspond to the classical and well‐characterized open and closed patterns of mesoscale cellular convection found over colder oceans (McCoy et al., 2017; Wood & Hartmann, 2006). Rather, shallow clouds in the trades appear organized in a less regular fashion, on scales ranging from 20 to 2,000 km. By inspecting 10 years of satellite imagery, Stevens et al. (2019) identified four recurrent patterns that they labeled “flowers”, “fish” , “gravel,” and “sugar.” In their classification, *sugar* consists of a dusting of very fine scale clouds with small vertical extension, *gravel*, of clouds organized along lines or arcs defining cells with intermediate granularity, sometimes looking like cold pools. *Fish*, were so named due to the appearance of a fishbone‐like skeletal network of clouds separated by well defined cloud‐free areas, and *flowers* denoted the presence of larger, seemingly more stratiform, cloud structures in the form of very large but dispersed closed cells. Examples of these four patterns are provided in Figure 1a.

Considerable daily and interannual variability in the appearance of the patterning (Stevens et al., 2019) offers an opportunity to investigate its co‐variability with large‐scale meteorological conditions. Even so, the relatively weak variability of the large‐scale environment (e.g., 90% of SST variations are weaker than 2 K) requires a record longer than the 10 winter seasons already classified. To access a longer record, we first attempt to identify the mesoscale patterns using an objective methodology and use this for exploring the co‐variability among patterns, their radiative effects, and the large‐scale environment in which they form. We do so by first demonstrating (section 2) that the four prominent patterns of cloud mesoscale organization pointed out by Stevens et al. (2019) project well onto a simple characterization of the spatial variability of infrared brightness temperatures measured from satellite. Then, in section 3, we investigate relationships between the four cloud patterns and the large‐scale environment in which they form. Finally, a summary of the main findings and a discussion of their implications for understanding low‐cloud feedbacks are presented in section 4.

## 2 Classification of Mesoscale Organization Patterns

We follow Stevens et al. (2019) by analyzing shallow clouds over the tropical Atlantic Ocean east of Barbados (48–58° W, 10–20° N) during the boreal winter (DJF, 1 December to 28 February), for the period from December 2000 through February 2019. Our analysis is, however, based on 3‐hourly infrared (11 μ m) brightness temperature ( $T_{b}$), gridded (0.07°) data from the GridSat‐B1 data set (Knapp et al., 2011). The calibration uncertainty of $T_{b}$ is less than 0.5 K and its stability better than 0.1 K/decade. To avoid situations obscured by occasional cirrus associated with deep convection over South America or within the ITCZ, we restrict our analysis to images for which the 25th percentile of $T_{b}$ is higher than 285 K. Pixels for which 280 K $\leq T_{b}\leq$ 290 K are associated with the presence of marine low‐cloud objects. This definition is purposefully conservative to exclude some of the thinnest cloud features that may correspond to evaporating cloud fragments. The threshold of 290 K corresponds to the temperature of cloud tops around 1 km, near the penetration depth of the most buoyant surface parcels (Stull, 1988; Vogel et al., 2019), and somewhat above the hazy layer of cumulus debris near cloud base. Finally, cloudy areas (or cloud objects) are defined through a nearest neighbor segmentation (e.g., Tobin et al., 2012). Each object is subsequently identified by its centroid, and area.

### 2.1 Organization Metrics

The population of cloud objects within the 10° × 10° area is characterized through a few metrics. Those include the total number of cloud objects N within the domain, the total fractional area A of the domain covered by shallow clouds, and a clustering measure, $I_{\text{org}}$, defined by Tompkins and Semie (2017) based on earlier work by Weger et al. (1992). $I_{\text{org}}$ compares the distribution of the nearest neighbor distances among the centroids of objects to that expected for a random distribution of objects. $I_{\text{org}}=0.5$ corresponds to randomly distributed centroids, while $I_{\text{org}}$ values significantly lower than 0.5 correspond to regular distributions, values higher than 0.5 correspond to “clustered” or “organized” distributions (supporting information Figure S1). These metrics are calculated for each 3‐hourly satellite image, and then daily‐mean values are computed.

The visual inspection of the day‐to‐day variability of cloud organizations suggests that at first order, the diversity of patterns can be characterized by only two metrics: the mean object size, $S=\frac{A}{N}\times 10^{4}$, which distinguishes patterns associated with a predominance of small or large cloud objects, and $I_{\text{org}}$. Over the period 2000–2019, S and $I_{\text{org}}$ exhibit a large variability with fairly continuous distributions (Figure 1b). By selecting situations that fall in the upper or lower terciles of both the S and $I_{\text{org}}$ distributions, we define four classes, or quadrants, that we refer to as A, B, C, and D, and which, as we show below, match well with the four cloud patterns identified by Stevens et al. (2019).

### 2.2 Cloud Patterns

Of the 900 images considered by Stevens et al. (2019), 815 were classified by at least one person as being dominated by one of the four patterns, and 337 were classified robustly (consistent classification by at least four people). To show that the cloud patterns are well delineated in the ( S, $I_{\text{org}}$) space, we consider all the robustly classified images falling into one of the four A‐B‐C‐D quadrants (154 images) and ask how frequently each pattern (flowers, fish, gravel, and sugar) fall into each quadrant. Figure 1c shows that the four quadrants of ( S, $I_{\text{org}}$) discriminate among the patterns reasonably well. “Flowers” occurs predominantly in Quadrant A, “fish” in B, “gravel” in C, and “sugar” in Quadrant D. This is consistent with the visual impression that the sugar and gravel patterns are mostly associated with small‐scale cloud features while the flowers and fish patterns are associated with more extended cloud features. According to the $I_{\text{org}}$ index, flowers and gravel patterns are associated with a close‐to‐random distribution of cloud features, while the fish and sugar patterns are associated with more clustered cloud objects.

The way in which the patterns distribute themselves in the ( S, $I_{\text{org}}$) space is largely intuitive, the one exception being the association of sugar with large values of $I_{\text{org}}$. As explained above, the cloud objects selected by the chosen brightness temperature thresholds do not correspond to the entire cloud population that exceeds the lifting condensation level, but only to the population of clouds whose top reaches the 290 K isotherm (about 1 km altitude). Sugar situations are characterized by the predominance of very fine scale clouds of very small vertical extent (Stevens et al., 2019). The rare active clouds that reach the 290 K isotherm often appear as isolated, so that their spatial distribution within the 10° × 10° area is characterized by a large $I_{\text{org}}$. Whereas flower and sugar are clearly separated classes, fish and gravel patterns show some overlap with flowers and sugar, respectively, an ambiguity that Stevens et al. (2019) also found in the visual classification.

Given the satisfactory correspondence between the visually identified patterns and the four ( S, $I_{\text{org}}$) quadrants, in the following we use the objective labeling to associate scenes distributed in Quadrants A, B, C, and D with “flowers”, “fish”, “gravel” and “sugar,” respectively. Adopting this methodology allows us to use the full GridSat‐B1 record to diagnose the daily occurrence of the four cloud patterns and their co‐variability with environmental conditions.

### 2.3 Robustness of the Classification

We test the robustness of the classification by repeating it using higher resolution MODIS (1 km) channel 31, and GOES‐16 (2 km) channel 13 brightness temperatures (Tables S1 and S2, Figures S2 and S3). MODIS provides twice daily data for the 2000–2019 period. From GOES‐16, we use 3‐hourly data for the last two winter seasons. The higher‐resolution data changes the number of cloud objects, the mean cloud object size, and the absolute value of $I_{\text{org}}$ (Figure S4). However, the day‐to‐day variability of the $I_{\text{org}}$, A and S metrics, correlates well among the different data sets (Table S3). Despite their very different spatial resolution (8 km vs. 2 km), GridSat and GOES‐16 classifications correlate best. For the case of $I_{\text{org}}$, the geostationary data (GOES and GridSat) correlate less well with MODIS, suggesting that $I_{\text{org}}$ may vary more with temporal sampling than it does with resolution.

The daily time series of mesoscale patterns (A, B, C, and D labels) determined from the upper and lower terciles of the MODIS data (Figure S2) correlate well with those identified using GridSat data (Table S3). The robustness of the classification provides further justification for our association of the upper and lower terciles of the ( S, $I_{\text{org}}$) distributions (the four quadrants) with “flowers”, “fish”, “gravel” and “sugar.”

## 3 Dependence of Patterns on the Large‐Scale Environment

To explore how large‐scale environmental conditions vary among the quadrants (patterns), we use 6‐hourly reanalyses of meteorological data as provided by the ERA‐interim product (Dee et al., 2011) for each DJF season from 2000 to 2019 and for several environmental variables: the SST, the near‐surface wind speed $V_{s}$, the zonal and meridional components of the surface wind $u_{s}$ and $v_{s}$, the zonal wind shear between 700 hPa and the surface, the large‐scale vertical velocity at 700 hPa, the lower‐tropospheric stability (LTS, defined as $\theta_{700}-\theta_{1,000}$, where θ is the potential temperature, Klein & Hartmann, 1993), and the estimated inversion strength (EIS, Wood & Bretherton, 2006), defined as $EIS=LTS-\Gamma_{m}^{850}(z_{700}-\text{LCL})$ where $\Gamma_{m}^{850}$ is the moist‐adiabatic potential temperature gradient at 850 hPa, $z_{700}$ is the height of the 700 hPa level, and LCL is the height of the lifting condensation level assuming a surface relative humidity of 80%. We also use layered free tropospheric relative humidity data from the Megha‐Tropiques satellite (Sivira et al., 2015). Each of these variables is computed as a daily‐mean average over the domain.

### 3.1 Day‐to‐Day Variability

**Figure 2:**
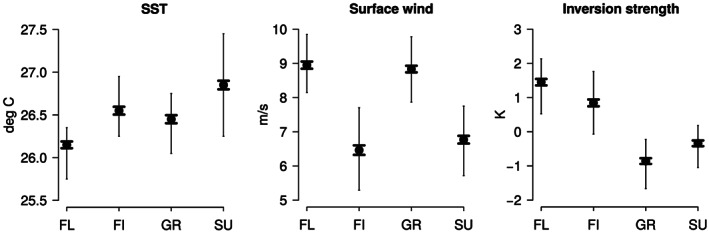
Large‐scale environmental conditions (daily‐mean SST, $V_{s}$, and EIS) composited over the 2000–2019 period as a function of the mesoscale cloud patterns (FL = flowers; FI = fish; GR = gravel; SU = sugar) inferred from GridSat data. Black markers indicate the mean of the distribution, thin vertical bars the range between the 25th and 75th percentile values, and thick lines ± the standard error on the mean.

To test whether different environmental conditions are associated with different patterns, a quadrant composite of each daily‐mean environmental variable is constructed. Most of the environmental variables considered do not differ significantly, or differ only marginaly, from one pattern to another (Figure S5). However a few variables, namely $V_{s}$ and EIS (equivalently LTS which correlates nearly perfectly [0.99] with EIS, but we adopt EIS because it generalizes to warmer climates more readily), were discriminating (Figure 2).

**Figure 3:**
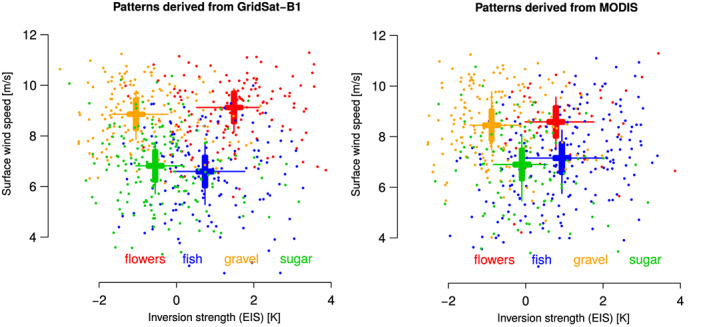
Scatter plot of daily‐mean values of EIS and near‐surface wind $V_{s}$ over 2000–2019. The mesoscale cloud patterns classified as flowers, fish, gravel, or sugar using (left) GridSat or (right) MODIS observations are indicated in colors. Also reported is the mean (EIS and $V_{s}$) value computed over the whole period for each cloud pattern. Thin bars indicate the 25th and 75th percentiles of the distributions, and thick bars indicate ± the standard error on the mean.

The analysis shows that “flowers” are associated with relatively cold SSTs, strong surface winds, and greater stability. “Fish” pattern were found over more moderate SSTs, weaker winds, and strong stability. “Gravel” was likewise associated with moderate SSTs but strong surface winds and low stability. “Sugar” prevailed over the warmest SSTs, when surface winds were weak and stability was low. It thus appears that EIS (or LTS, Figure S6) best discriminates the patterns with small versus large S: the patterns associated with large cloud objects (flowers and fish) predominantly occur in situations with a more stable lower troposphere. This is consistent with the expectation that larger stratiform cloud fields are to be expected in situations with enhanced stability (Klein & Hartmann, 1993; Wood & Bretherton, 2006). $V_{s}$ best discriminates the type of convective organization ( $I_{\text{org}}$): Random to more regular organizations of cloud centroids (flowers and gravel, both associated with low $I_{\text{org}}$ values) tend to occur when the trade winds are strong ( $V_{s}\geq 8m\;s$), while the more “organized” distributions (sugar and fish) tend to occur when the trades are weaker. Overall, the cloud patterns that correspond to the most contrasted S and $I_{\text{org}}$ metrics, namely, the flowers and sugar patterns, are those that occur in the most contrasted environments. Repeating this analysis using the MODIS classification (Figure 3) leads to similar conclusions.

### 3.2 Interannual Variability

**Figure 4:**
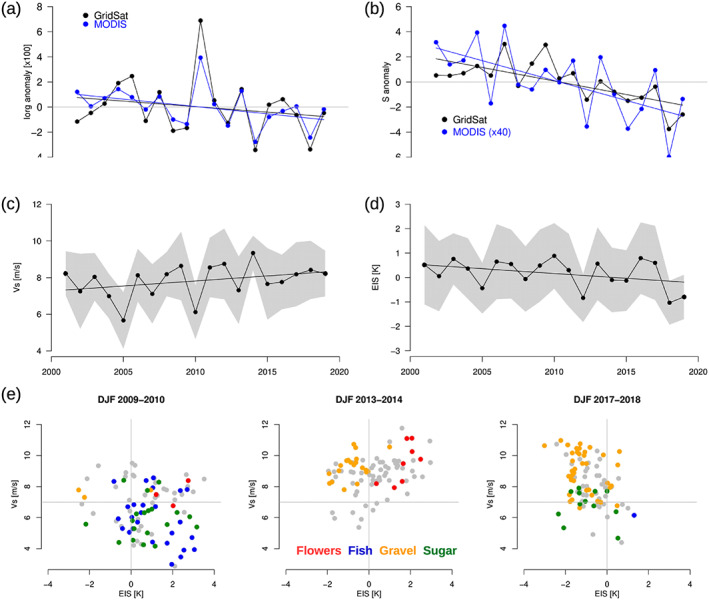
Interannual anomalies of (a) the organization index (I ${}_{org}$) and (b) the mean cloud object size S computed from GridSat or MODIS observations over the period 2000–2019 during DJF (the correlation between GridSat and MODIS time series is 0.90 for I ${}_{org}$ and 0.78 for S). Interannual evolution of (c) $V_{s}$ and (d) EIS derived from ERA interim for the same period. Note that in (a–d), the year is defined by the January–February months of the DJF season (e.g., 2010 corresponds to December 2009–February 2010). The shading represents ±1 standard deviation of daily‐mean values around the DJF mean. (e) Examples, for a few DJF seasons, of the daily cloud patterns identified from GridSat data represented as a function of the daily (EIS and $V_{s}$) conditions of that season (the gray lines are just visual guides).

The analysis of daily variability was extended to explore interannual variability by comparing year‐to‐year variations of DJF means, each DJF mean being computed by filtering out the days obscured by upper‐level clouds or without cloud pattern classification. Once again, the variability of $I_{\text{org}}$ and S derived from GridSat‐B1 and MODIS data sets are consistent with each other (Figures 4a and 4b). The interannual relationships between these metrics and environmental conditions are also consistent with those found at the daily timescale: $I_{\text{org}}$ anomalies exhibit a negative correlation with $V_{s}$ anomalies, and S anomalies exhibit a positive correlation with EIS anomalies (Figures 4c and 4d, Table S4). On the other hand, the correlation between these metrics and SST (whose time evolution is shown in Figure S7) is not significant at the interannual timescale.

For each season, the relative prominence of the four patterns is consistent with the $V_{s}$ and stability anomalies of that season (Figure 4e). For instance, the 2009–2010 DJF season which was characterized by an anomalously weak $V_{s}$ (Figure 4c) and an anomalously strong stability (Figure 4d) was associated with a predominance of “fish.” In contrast, the 2013–2014 DJF was associated with a very strong surface wind and a predominance of “gravel” and “flowers” patterns, while the 2017–2018 DJF was associated with weak stability and was mostly associated with “gravel” and “sugar.” The association between cloud patterns and large‐scale environmental conditions (as characterized by $V_{s}$ and EIS) pointed out at the daily timescale is thus able to also explain the year‐to‐year variations of the spatial organization metrics and the predominance of a specific mesoscale cloud pattern (Figure S8).

## 4 Summary and Discussion

Stevens et al. (2019) showed, based on a visual and thus subjective classification, that the tropical western Atlantic during boreal winter is associated with four prominent mesoscale patterns of shallow convection. The present study shows that these patterns can be objectively identified based on the size and degree of clustering of segmented cloud objects as identified from infrared brightness temperatures. The classification is largely insensitive to the spatial resolution of the brightness temperature data: GridSat data with a resolution of 8 km and MODIS data with a resolution of 1 km lead to very similar classifications.

The analysis of daily and interannual variations shows that the relative occurrence of the different cloud patterns correlates strongly with two environmental factors: the strength of the near‐surface wind speed and the strength of the lower‐tropospheric stability (Figures 3 and S2). Flowers tend to occur in windy ( $V_{s}$ > 8 m s ${}^{-1}$) and stable environments ( EIS > 0.5 K), while sugar tends to occur in calm ( $V_{s}$ < 8 m s ${}^{-1}$) and unstable environments ( EIS<0.5K). Fish appears to prefer calm and stable environments, while gravel tends to occur in windy and unstable environments. These relationships beg a physical explanation. For this purpose, data from the forthcoming 2020 EUREC^4^A (*Elucidating the role of cloud‐circulation coupling in climate*) field campaign should be well suited (Bony et al., 2017). With its large complement of air and sea‐going vessels in the same study region as examined here, EUREC^4^A will not only quantify the relationship between cloud patterns and large‐scale variables but also the circulation systems that connect the two. These measurements should thus also help determine how much the mesoscale organization of shallow convection has to be considered if one wants to understand and predict the response of shallow clouds to changes in environmental conditions.

**Figure 5:**
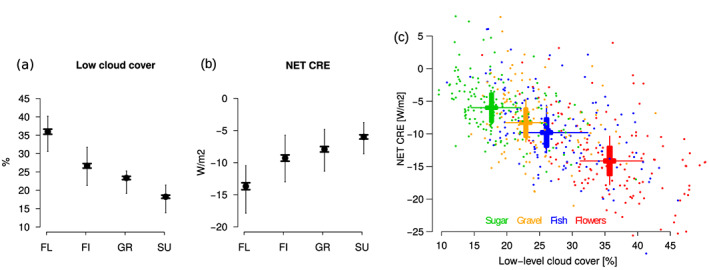
(a) and (b) same as Figure 2 but for the low‐cloud amount derived from MODIS cloud products and the NET cloud‐radiative effect derived from CERES observations. (c) Same as Figure 3 but for daily‐mean values of NET CRE and low‐level cloud amount.

A closely related question is whether the mesoscale organization of shallow convection matters for cloud‐radiative effects. To shed light on this issue, we used daily estimates of top‐of‐atmosphere radiative fluxes and cloud products from the CERES (Clouds and the Earth's Radiant Energy System) geostationary enhanced temporally interpolated data set (Wielicki et al., 1996), along with low‐cloud amount retrievals provided as part of the same data set for the period 2001–2017. Low‐level cloud amount varies by a factor of two across the different patterns, and the net CRE associated with “flowers” is about double that of the sugar pattern (Figure 5). At first order, the CRE (dominated by its shortwave component) varies linearly with the low‐cloud amount (Klein & Hartmann, 1993), so that radiative differences across the patterns are related to differences in the low‐cloud amount. However, unlike what has been found for other types of mesoscale organizations of marine low clouds (McCoy et al., 2017), *for a given low‐cloud amount* we do not notice significant radiative difference among patterns. It suggests that over the western tropical Atlantic, changes in the mesoscale organization of trade‐wind cumuli primarily affect the top‐of‐atmosphere radiation budget through associated changes in the low‐level cloud amount.

Could this tight relationship between convective organization and low‐cloud amount, or CRE, imply that changes in cloud organization have the potential to influence cloud‐radiative feedbacks? The large‐scale environment in which the trade‐wind cumuli form might change under global warming. Climate models predict EIS increases over the western tropical Atlantic as the planet warms (Qu et al., 2015). On the other hand, the change in $V_{s}$ remains uncertain, partly because the geographical pattern of surface warming can act against the anticipated slow‐down of the large‐scale circulation (Ma et al., 2016). Indeed, in climate change experiments run with the IPSL climate model (Dufresne et al., 2013), EIS always increases with global warming over the tropical western Atlantic (by 0.1 to 0.7 K K ${}^{-1}$ depending on the type of experiment and model version), whereas $V_{s}$ does not change in a robust fashion. Assuming that $V_{s}$ and EIS remain the main controlling factors of the mesoscale organization of shallow clouds in a perturbed climate, these projections would suggest a more frequent occurrence of fish or flower at the expense of sugar or gravel with global warming and thus a larger cloud fraction. This is in conflict with the prevailing idea, based on models which do not account for mesoscale organization, that low‐cloud amount will reduce in response to rising SST (Klein et al., 2017). In our analysis, SST does not appear to be a strong controlling factor of the cloud mesoscale organization on daily and interannual timescales (Table S2), but it remains an open question whether it could play a bigger role in climate change. In either case, better understanding the extent to which the mesoscale patterning of clouds affects their response to warming appears relevant to establishing confidence in how clouds respond to warming as a whole.

Future investigations of this issue using numerical models that predict explicitly these different cloud patterns and are able to reproduce the relationships discussed in this paper should help determine how much the cloud organization is sensitive to SST, and how much it could affect the magnitude and even maybe the sign of the change in low‐cloud amount. This should fill an important gap in our understanding and our assessment of low‐cloud feedbacks under climate change.

## Supporting information



2019GL085988‐sup‐0001‐Text_SI‐S01.pdf
