# Supplementary material for: Sugar, Gravel, Fish, and Flowers: Dependence of Mesoscale Patterns of Trade‐Wind Clouds on Environmental Conditions
Source: Geophys Res Lett. 2020 Mar 26;47(7):e2019GL085988. doi: 10.1029/2019GL085988 (PMC7375147; doi:10.1029/2019GL085988)
Supplement: Supplementary file 1 — 2019GL085988‐sup‐0001‐Text_SI‐S01.pdf [file GRL-47-e2019GL085988-s001.pdf]

Supporting Information for "Sugar, Gravel, Fish and Flowers: Dependence of Mesoscale Patterns of Trade-wind Clouds on Environmental Conditions"

Sandrine Bony[1], Hauke Schulz[2], Jessica Vial[1]and Bjorn Stevens[2]

[1]LMD/IPSL, CNRS, Sorbonne University, Paris, France

[2]Max Planck Institute for Meteorology, Hamburg, Germany

Contents of this file

1. Tables S1 and S4
2. Figures S1 to S8

Corresponding author: Sandrine Bony, LMD/IPSL, CNRS, Sorbonne University, mailbox 99, 4 Place Jussieu, 75252 Paris cedex 05, France. (sandrine.bony@lmd.jussieu.fr)

Introduction In this Supporting Information, we provide a table reporting statistics about the number of satellite images used for the cloud pattern classification (Tables S1 and S2), correlations between the cloud organization metrics computed with different satellite datasets (Table S3), and a table comparing the daily and interannual correlations between the cloud organization metrics and environmental conditions (Table S4). We also provide a figure explaining how the I_{org} index is computed (Figure S1), two figures comparing the brightness temperature fields derived from different satellite datatsets for different types of mesoscale patterns (Figures S2 and S3), a figure showing how the shallow convective organization patterns can be defined using different satellite datasets (Figure S4), two figures showing different environmental variables composited by the four mesoscale patterns of trade-wind cloudiness (Figures S5 and S6), a figure showing the interannual variability of the sea surface temperature over 2000-2019 (Figure S7) and a figure showing how the different cloud patterns relate to the surface wind speed and the lower-tropospheric stability for each DJF season of the 2000-2019 period (Figure S8).

Table S1. Frequency of occurrence (expressed in absolute and relative values over the 19 DJF seasons of 2000-2019) of days associated with low clouds, non-low clouds or missing images (note that MODIS images are disregarded when the swath data do not fill completely the 58W-48W,10N-20N area).

		Total	Low clouds	Non low clouds	Missing
GridSat	N	1714	1343	312	59
(Dec2000-Feb2019)	%	100	78.3	18.2	3.5
MODIS	N	1714	1092	317	305
(Dec2000-Feb2019)	%	100	63.7	18.5	17.8
GOES-16	N	180	151	16	13
(Dec2017-Feb2019)	%	100	83.9	8.9	7.2

Table S2. Frequency of occurrence (expressed in absolute and relative values) of low-cloud situations whose mesoscale pattern was classified as Flowers, Fish, Gravel or Sugar, or not classified (because the I_{org} or S metrics do not belong to the first or third terciles of their distribution).

		Low clouds	Non classified	Classified	Flowers	Fish	Gravel	Sugar
GridSat	N	1343	746	597	142	134	152	169
(Dec2000-Feb2019)	%	100	55.5	44.5	10.6	10.0	11.3	12.6
MODIS	N	1092	581	511	48	194	173	96
(Dec2000-Feb2019)	%	100	53.2	46.8	4.4	17.8	15.8	8.8
GOES-16	N	151	82	69	5	23	27	14
(Dec2017-Feb2019)	%	100	54.3	45.7	3.3	15.2	17.9	9.3

Table S3. Linear correlation coefficients between the daily-mean metrics calculated with different satellite datasets (the convective organization index I_{org}, the mean cloud object size S, and the total area covered by cloud objects A): GridSat-1B (8 km resolution, 3-hourly), GOES16 (2 km resolution, 3-hourly), MODIS (1 km resolution, twice-per-day). The last line reports the % of images for which the daily mesoscale pattern (Flowers, Fish, Gravel or Sugar) is classified consistently by two different datasets.

	R(GridSat,MODIS) (Dec2000-Feb2019)	R(GridSat,MODIS) (Dec2017-Feb2019)	R(GridSat,GOES16) (Dec2017-Feb2019)	R(MODIS,GOES16) (Dec2017-Feb2019)
I_{org}	0.61	0.65	0.84	0.61
S	0.76	0.89	0.88	0.90
A	0.87	0.90	0.97	0.92
Agreement (%)	76	82	70	90

Table S4. Linear correlation coefficients of I_{org} and S (computed either from GridSat-B1 or MODIS data) with V_s, EIS and SST (derived from ERA interim reanalyses). Correlations are computed for day-to-day variations within DJF, and interannual variations of DJF means. Numbers are bracketed when the correlation is not statistically significant (p-value larger than 0.01).

	Daily			Interannual		
	V_s	EIS	SST	V_s	EIS	SST
I_{org} (GridSat)	-0.57	(0.03)	0.23	-0.86	(0.41)	(0.55)
I_{org} (MODIS)	-0.43	0.25	0.10	-0.78	(0.54)	(0.36)
S (GridSat)	(0.01)	0.54	-0.23	(-0.12)	0.48	(-0.14)
S (MODIS)	(-0.05)	0.45	-0.10	(-0.09)	0.60	(-0.12)

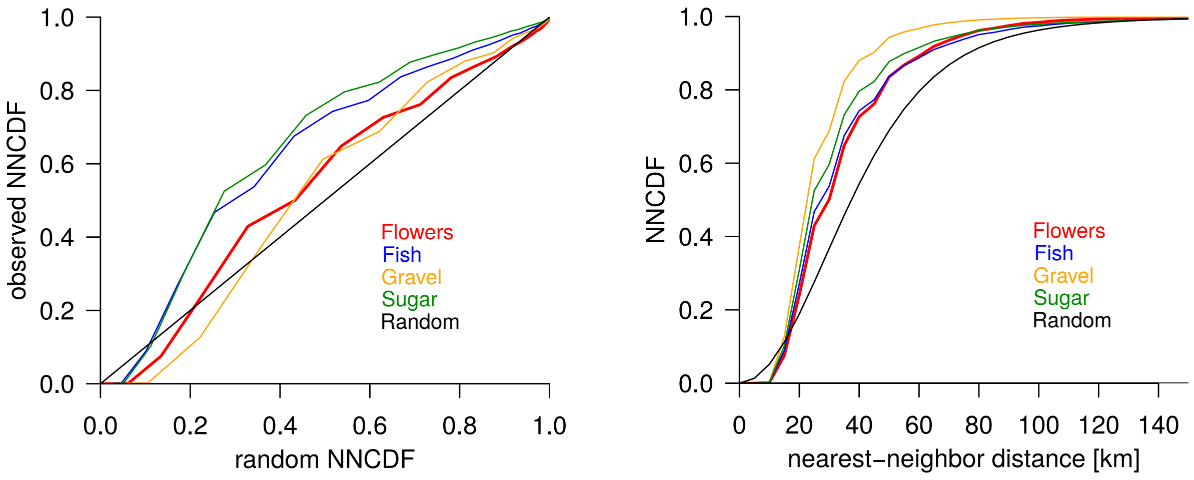

Figure S1. The I_{org} index compares the cumulative density function of the nearest neighbor distances among the centroids of cloud objects inferred from observations (NNCDF) to that expected for a random distribution of objects. (left) NNCDF of the cloud objects detected from GridSat observations of infrared brightness temperature for each type of cloud pattern (computed over the 2000-2019 period). Most of the nearest-neighbor distances range from 10 to 100 km. (right) Relationship (based on the whole GridSat dataset) between the observed and random NNCDFs for each cloud pattern; the I_{org} index is computed by integrating the area under the observed NNCDF (I_{org} = 0.5 corresponds to a random distribution).

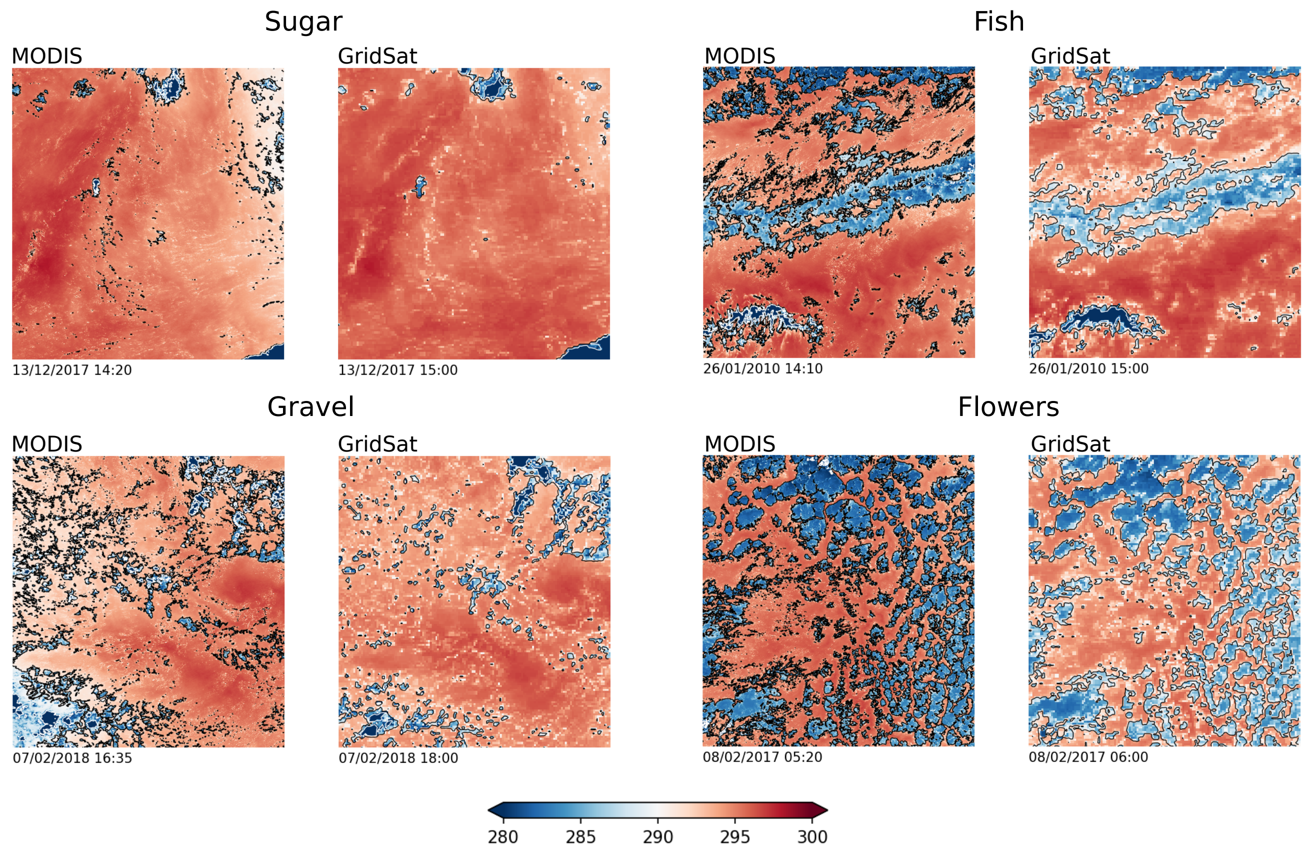

Figure S2. Sugar, Fish, Gravel and Flowers cloud mesoscale patterns as seen from the instantaneous brightness temperature (T_b) field derived from the MODIS or GridSat dataset over (58W-48W; 10N-20N). The spatial resolution of MODIS is 1 km while that of GridSat is about 8 km. The 280 K and 290 K levels used to identify cloud objects are shown by white and black contour lines, respectively (cloud objects appear in blue).

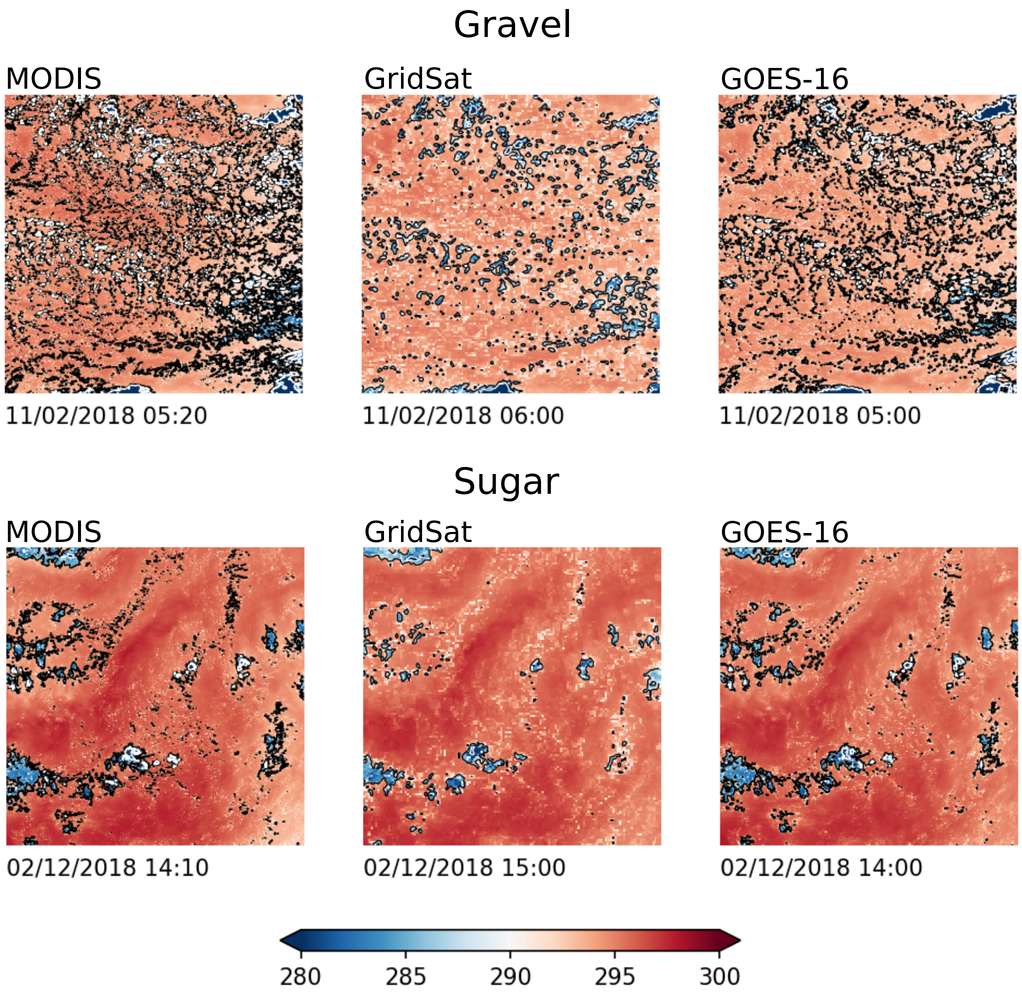

Figure S3. Same as Figure S2 but for the Gravel and Sugar patterns seen from the instantaneous brightness temperature (T_b) field derived from the MODIS, GridSat or GOES-16 dataset over (58W-48W; 10N-20N). The spatial resolution of MODIS, GridSat and GOES-16 are 1 km, 8 km and 2 km, respectively. The 280 K and 290 K levels used to identify cloud objects are shown by white and black contour lines, respectively (cloud objects appear in blue).

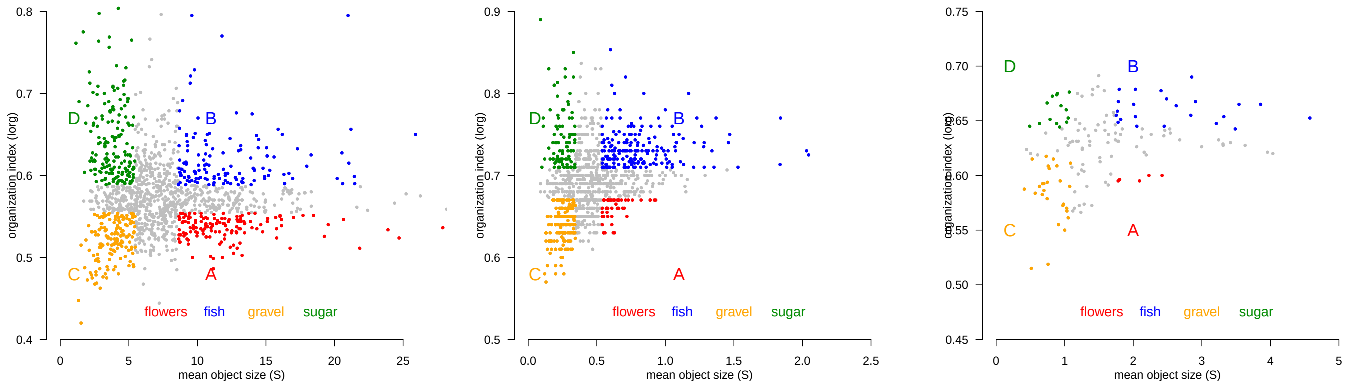

Figure S4. Definition of the shallow convective organization patterns using the first and last terciles of the convective organization index (I_{org}) and the mean cloud object size (S) derived from (left) GridSat-B1, (middle) MODIS and (right) GOES-16 datasets. The GridSat-B1, MODIS and GOES-16 datasets have a spatial resolution of about 8 km, 1 km and 2 km, respectively. GridSat-B1 and MODIS data are available from December 2000 to February 2018, while GOES-16 data are available from December 2017 to February 2019 only. By analogy with Figure 1, the quadrants A, B, C and D and referred to as Flowers, Fish, Gravel and Sugar.

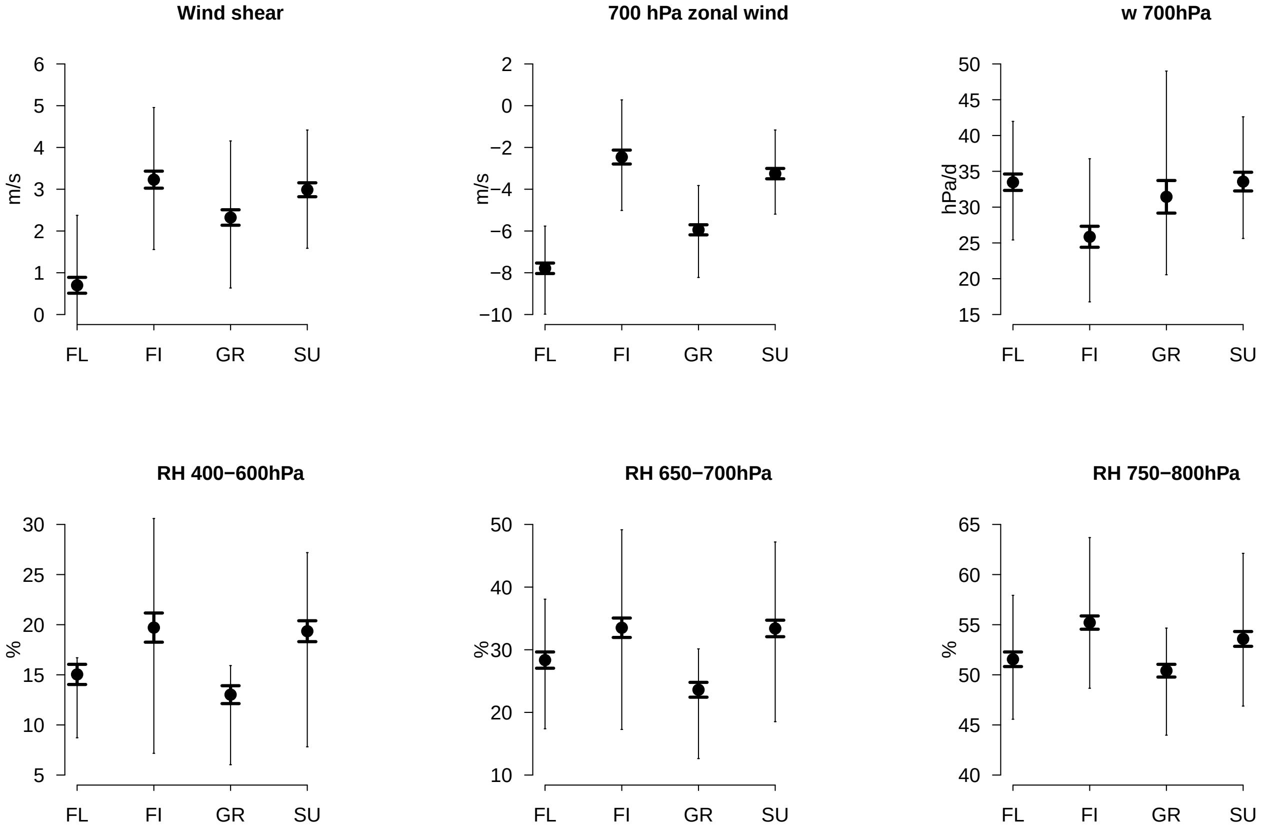

Figure S5. Large-scale environmental conditions (daily-mean wind shear, zonal wind at 700 hPa, large-scale pressure vertical velocity at 700 hPa derived from ERA interim reanalyses during the DJF seasons of 2000-2019, daily-mean relative humidity in the layers 400-600 hPa, 650-700 hPa and 750-800 hPa derived from the Megha-Tropiques satellite during the DJF seasons of 2012-2018) composited as a function of the mesoscale cloud patterns (FL=Flowers; FI=Fish; GR=Gravel; SU=Sugar) inferred from GridSat data. Black markers indicate the mean of the distribution, thin vertical bars the range between the 25th and 75th percentile values, and thick lines $\pm$ the standard error on the mean.

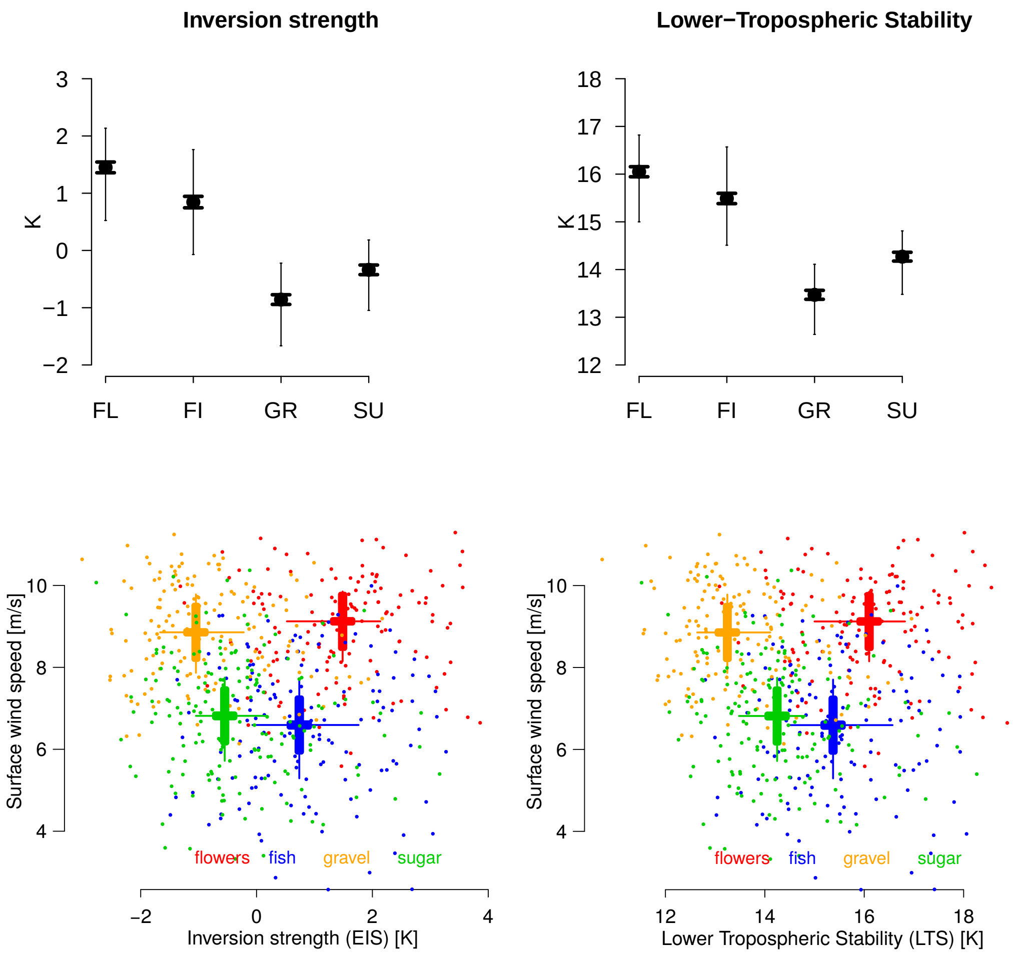

Figure S6. Comparison of the dependence of cloud patterns on two measures of the tropospheric stability: the Estimated Inversion Strength (EIS) and the Lower Tropospheric Stability (LTS). See Figures 2 and 3 for a more detailed explanation of the different symbols (EIS panels are reproduced from Figures 2 and 3).

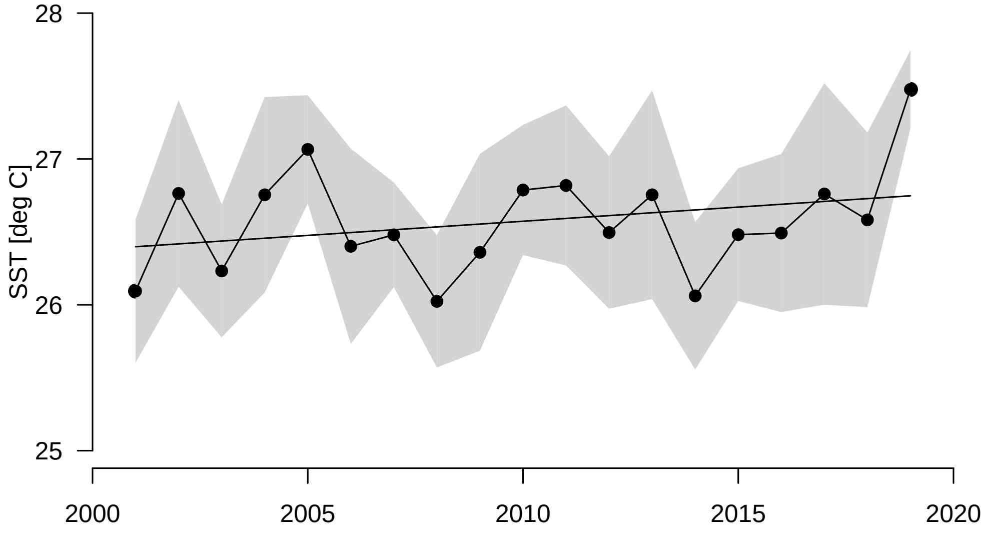

Figure S7. Interannual evolution of the sea surface temperature derived from ERA interim over 2000-2019. The shading represents ± one standard deviation of daily-mean values around the DJF mean.

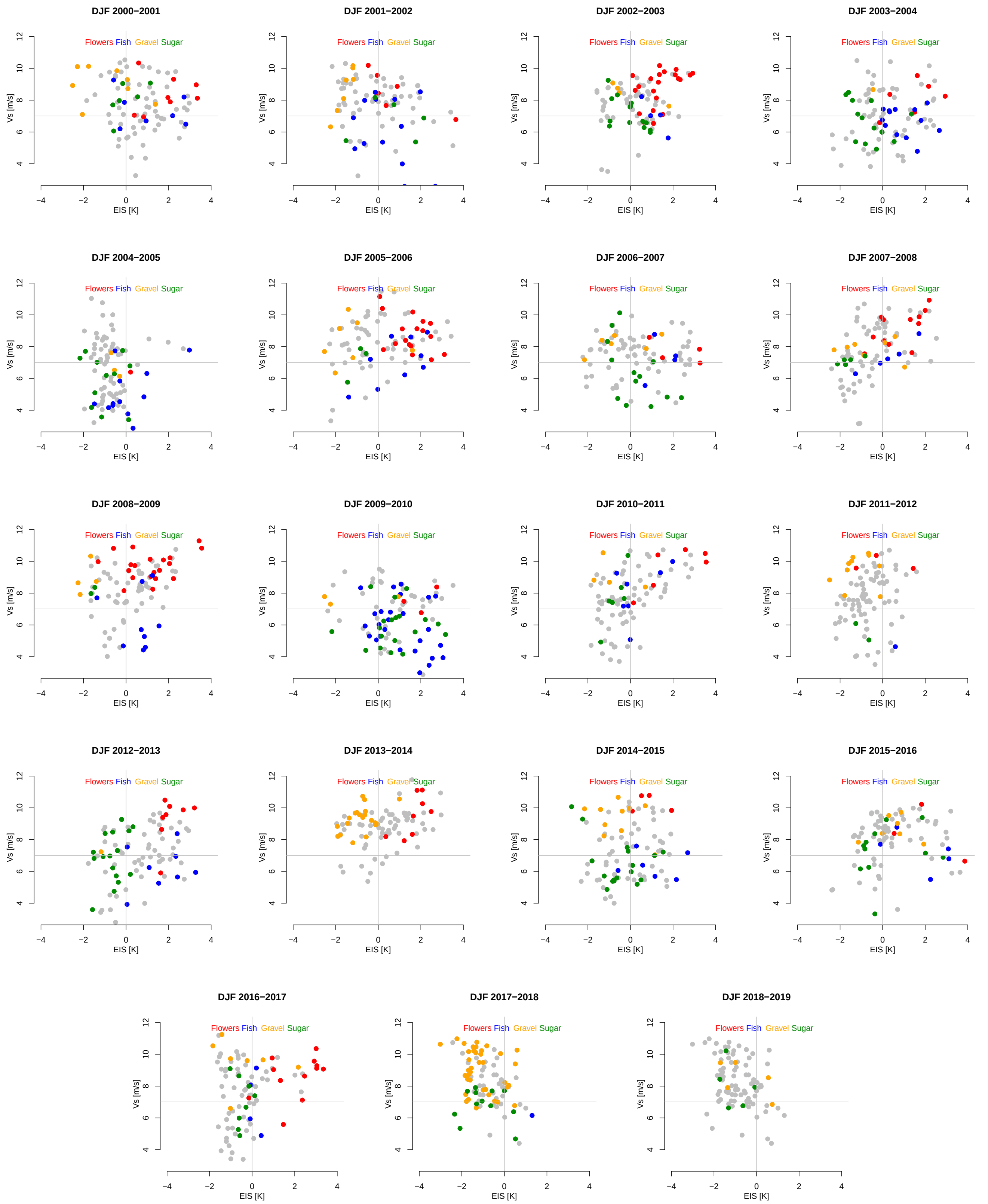

Figure S8. For each DJF season: cloud patterns identified from GridSat data represented as a function of the daily (EIS, V_s) conditions of that season. Each pattern is associated with a different color. Grey markers correspond to days for which no cloud pattern was identified, either because the area was not predominantly covered by low-level clouds, or because the (S, I_{org}) metrics were outside the A, B, C, D quadrants defining the different patterns. The grey lines at EIS = 0 K and V_s = 7 m.s^{-1} are just visual guides.
